# Dynamic changes in extracellular vesicle-associated miRNAs elicited by ultrasound in inflammatory bowel disease patients

**DOI:** 10.1038/s41598-024-61532-2

**Published:** 2024-05-13

**Authors:** Florian Tran, Alena Scharmacher, Nathan Baran, Neha Mishra, Marek Wozny, Samuel Pineda Chavez, Archana Bhardwaj, Sophia Hinz, Simonas Juzenas, Joana P. Bernardes, Laura Katharina Sievers, Matthias Lessing, Konrad Aden, Arne Lassen, Arne Bergfeld, Hauke Jann Weber, Lennart Neas, Stefania Vetrano, Stefan Schreiber, Philip Rosenstiel

**Affiliations:** 1 Institute of Clinical Molecular Biology, University Medical Center Schleswig-Holstein, Christian Albrecht University Kiel, Campus Kiel, Rosalind-Franklin-Strasse 12, 24105 Kiel, Germany; 2 Department of Internal Medicine I, University Medical Center Schleswig-Holstein, 24105 Kiel, Germany; 3 Department of Biomedical Sciences, Humanitas University, 20072 Pieve Emanuele, Italy; 4 Institute of Biotechnology, Life Science Centre, Vilnius University, Vilnius, Lithuania; 5 Department of Gastroenterology, Asklepios Westklinikum, 22559 Hamburg, Germany; 6 Institute of Infection Medicine, University Medical Center Schleswig-Holstein, Christian Albrecht University Kiel, 24105 Kiel, Germany; 7 Department of Internal Medicine III, University Medical Center Schleswig-Holstein, 24105 Kiel, Germany; 8 IBD Unit, Department of Gastroenterology, IRCCS Humanitas Research Hospital, 20089 Rozzano, Italy

**Keywords:** Inflammatory bowel disease, Liquid biopsies, Extracellular vesicles, Sonography, Biomarker discovery, Inflammatory bowel disease, Crohn's disease, Ulcerative colitis, Gene expression, Diagnostic markers

## Abstract

Blood-based biomarkers that reliably indicate disease activity in the intestinal tract are an important unmet need in the management of patients with IBD. Extracellular vesicles (EVs) are cell-derived membranous microparticles, which reflect the cellular and functional state of their site of site of origin. As ultrasound waves may lead to molecular shifts of EV contents, we hypothesized that application of ultrasound waves on inflamed intestinal tissue in IBD may amplify the inflammation-specific molecular shifts in EVs like altered EV-miRNA expression, which in turn can be detected in the peripheral blood. 26 patients with IBD were included in the prospective clinical study. Serum samples were collected before and 30 min after diagnostic transabdominal ultrasound. Differential miRNA expression was analyzed by sequencing. Candidate inducible EV-miRNAs were functionally assessed in vitro by transfection of miRNA mimics and qPCR of predicted target genes. Serum EV-miRNA concentration at baseline correlated with disease severity, as determined by clinical activity scores and sonographic findings. Three miRNAs (miR-942-5p, mir-5588, mir-3195) were significantly induced by sonography. Among the significantly regulated EV-miRNAs, miR-942-5p was strongly induced in higher grade intestinal inflammation and correlated with clinical activity in Crohn’s disease. Prediction of target regulation and transfection of miRNA mimics inferred a role of this EV-miRNA in regulating barrier function in inflammation. Induction of mir-5588 and mir-3195 did not correlate with inflammation grade. This proof-of-concept trial highlights the principle of induced molecular shifts in EVs from inflamed tissue through transabdominal ultrasound. These inducible EVs and their molecular cargo like miRNA could become novel biomarkers for intestinal inflammation in IBD.

## Introduction

Inflammatory bowel disease (IBD), with its two main entities Crohn’s disease (CD) and ulcerative colitis (UC) is characterized by chronic inflammation of the intestine. There is a high degree of heterogeneity of the extent and localization of the inflamed gut tissue, which can range from small superficial lesions to entire segments of transmural inflammation. The development of targeted therapies in IBD has led to a need to better define therapeutic outcomes. The current ‘treat-to-target’ strategies require objective indicators of disease activity and of disease control subsequent to a given therapy. Ileo-colonoscopy is the gold standard for the assessment of disease activity with the possibility of diagnostic histopathology from biopsies^1^. Endoscopic evaluation of the mucosa, however, has natural limitations in CD, where it may not reach the site of inflammation. Although the risk of complications is small, it also represents a significant adverse experience for any IBD patient and imposes a financial burden on healthcare systems. Intestinal ultrasound (US) imaging has become a powerful non-invasive tool to monitor intestinal disease activity, particularly in CD, and is widely available and broadly used by healthcare professionals^2,3^. The downside of this method, though, is the lack of direct access to tissue material for histopathologic or biomarker evaluation. C-reactive protein (CRP) and fecal calprotectin are widely used and established disease activity markers in IBD, which are obtained from easily accessible material. Yet, they have significant weaknesses including their lack of disease specificity and sensitivity^4^.

To overcome the limitations of each of the abovementioned assessment tools, the concept of precise liquid biopsies that reflect tissue pathology and are detectable in peripheral blood arose. In this context, extracellular vesicles (EVs) were brought into the focus as they are a heterogenous group comprising exosomes, ectosomes, apoptotic bodies and further vesicle types with a variety of proposed physiological functions^5^. EVs contain several classes of biomolecules on the surface and as luminal cargo and resemble their parent tissue cells through distinct molecular features^6^. In cancer, evidence suggests that EVs play a crucial role in facilitating long-range effects, such as distant metastasis and systemic immune modulation, thereby contributing to the progression and dissemination of cancer cells throughout the body^7,8^.

Previous work on their role in inflammation demonstrated that functional inflammasome complexes are transferred from donor to effector cells by EVs, while the composition of the cargo changed upon inflammatory stimuli^9^. Recent in vitro studies showed that application of low-intensity pulsed ultrasound (LIPUS) on bone marrow mesenchymal stem cells increased their secretion of EVs and altered the EV molecular cargo^10^. While other molecular cargo of EVs like surface proteins or lipids have potential applications as biomarkers, microRNAs (miRNAs) derived from EVs offer the unique feature as long-range genomic regulators in the effector cells^11,12^, which have been linked to the pathogenesis^13^ of IBD and as disease biomarkers^14^. Although diagnostic US imaging uses high-frequency sound pulses that differ from LIPUS for allowing image reconstruction without tissue aberrations, US waves provide forces on tissue that albeit weak, can perturb tissue-resident cells to transiently alter molecular expression profiles and EV secretion.

Here, we hypothesized that conventional transabdominal US stimulates the release of distinct EVs and/or specific molecular EV cargo from inflamed intestinal tissue. Induced molecular features of EVs could represent the inflammatory state of the intestine more precisely, which may thus serve as an inducible and tissue-specific circulating biomarker for monitoring disease activity.

In this proof-of-principle prospective study, we performed profiling of EV cargo miRNAs before and after a standardized transabdominal US procedure in a cohort of twenty-six IBD patients. We screened for induced miRNA candidates, which were associated with intestinal inflammation. We identified three differentially expressed miRNAs in the peripheral blood that were dynamically upregulated after US application and assessed their function.

## Results

### Detection of increased EV-miRNA after intestinal ultrasound

**Figure 1:**
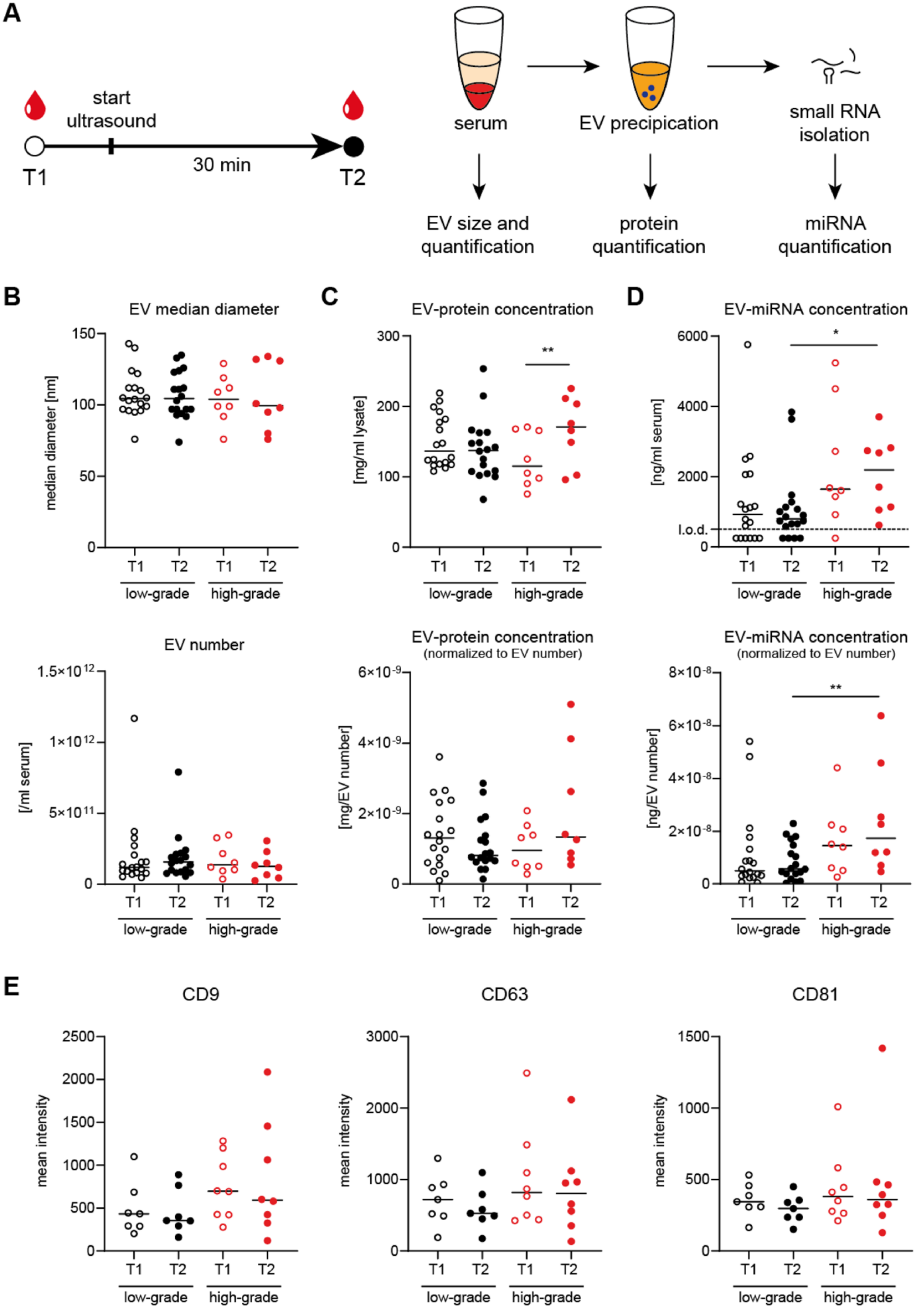
Detection of proteins and miRNA from serum EVs before and after intestinal US. (**A**) Schematic study design and sample processing pipeline. A total of *n* = 26 patients (*n* = 18 low-grade, *n* = 8 high-grade inflammation) with each two timepoints were included in the downstream analysis in (**B**–**D**). For (**E**), a fraction of *n* = 15 patients (*n* = 7 low-grade, *n* = 8 high-grade inflammation) with each two timepoints were included. (**B**) EV count and median diameter (normalized to serum volume) stratified by time point and degree of acute intestinal inflammation as assessed by intestinal US. No statistically significant differences in all comparisons. (**C**) EV protein concentrations (normalized to serum volume or EV count) stratified by timepoint and degree of acute intestinal inflammation. Only in the “high grade” inflammation group intestinal US increased levels of EV protein concentration (paired *t*-test; **P < 0.01). (**D**) EV-miRNA concentrations (normalized to serum volume or EV count) stratified by timepoint and degree of acute intestinal inflammation. EV-miRNA concentration after US was increased in the “high grade” versus “low grade” inflammation group (unpaired *t*-test; *P < 0.05, **P < 0.01). (**E**) Mean intensities of EV-surface protein markers CD9, CD63 and CD81 as quantified by flow cytometry, stratified by timepoint and degree of acute intestinal inflammation. No significant differences in all comparisons.

First, we examined whether intestinal US increases levels of EV-associated biomolecules (Fig. 1A). Since the biological effects of US on the cells are weak and transients, we quantified EVs levels after 30 min of the procedure as proposed peak of EV secretion^15^. Numbers and size of serum EVs were not significantly altered in high-grade vs. low-grade inflammation or between timepoints (Fig. 1B). Quantification of EV-specific surface markers CD9, CD63 and CD91 suggested similar composition of EVs independent from disease severity and time point (Fig. 1E). All further quantified protein markers were not significantly altered (data not shown).

We showed that overall protein concentrations of purified EVs were increased after the US procedure (T2) versus baseline (T1) in the high-grade inflammation group (*P* = 0.0086 in paired *t*-test, normalized to EV count: *P* = 0.0830) while no temporal changes were observed in the low-grade inflammation group (Fig. 1C). EV-miRNA concentrations were highly variable between individuals, and concentrations were increased in the high vs. low grade inflammation group after intestinal US at T2 (*P* = 0.0392 in unpaired *t*-test, normalized to EV count: *P* = 0.0289) (Fig. 1D). Taken together, these results hinted towards altered EV-miRNA content in the high-grade inflammation group after intestinal US rather than general enhanced release of EVs.

### Differential EV-miRNA expression after intestinal ultrasound

**Figure 2:**
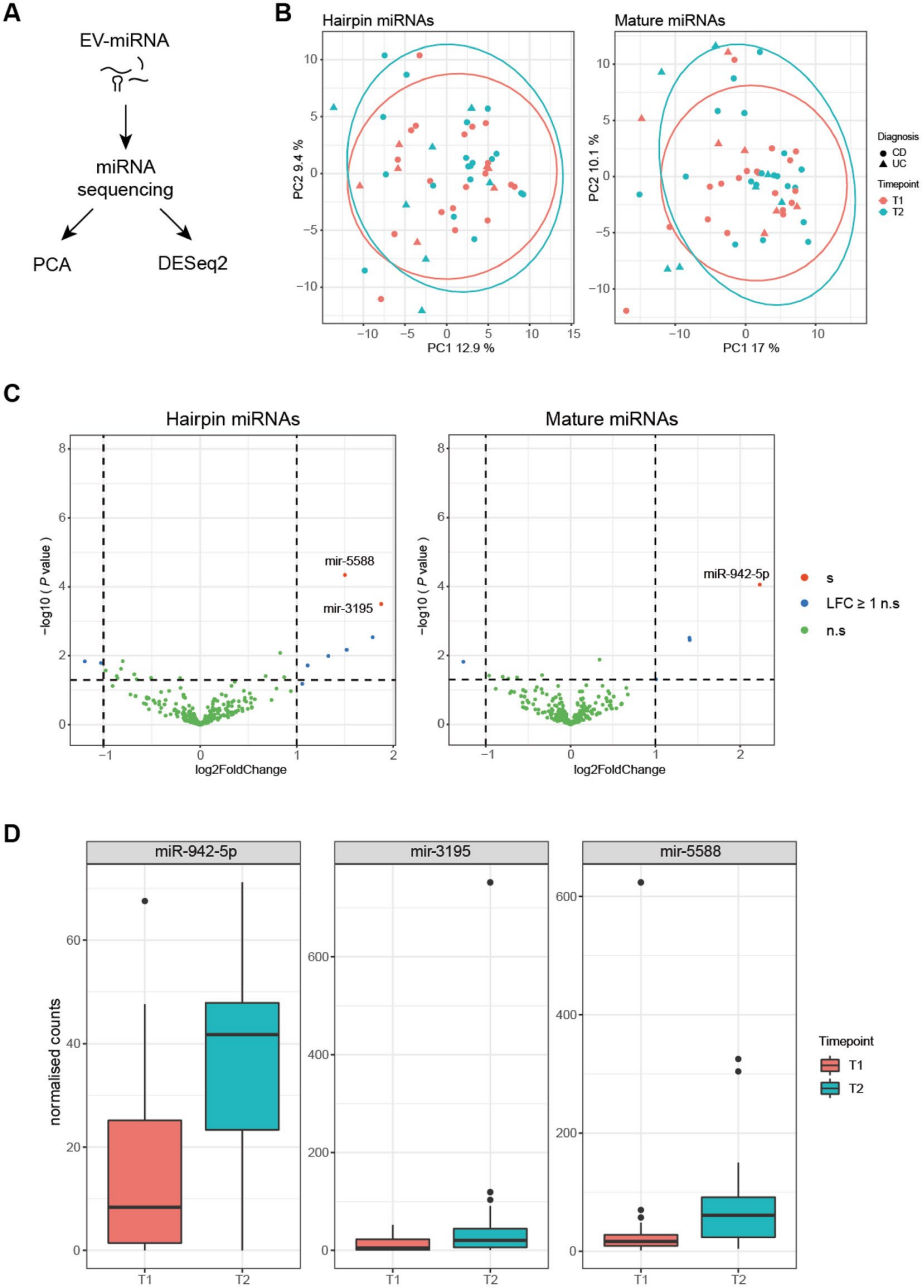
Differential EV-miRNA analysis between the two timepoints in the study cohort. (**A**) Schematic overview of the bioinformatical analysis workflow. (**B**) PCA plots of annotated miRNA, separated by mature miRNA (*left*) and hairpin miRNA (*right*). Ellipses represent 95% confidence intervals of each group. red: T1; blue: T2; circles: CD patients, triangles: UC patients. (**C**) Volcano plots of differentially expressed miRNA between timepoints. X-axis: Log2FoldChange; Y-axis: Log10 *P* value. Each dot represents one miRNA. Green: not differentially expressed, blue: |Log2FoldChange| > 1 but no significant differential expression, red: |Log2FoldChange| > 1 and significant differential expression. (**D**) Normalized counts of miR-942-5p, mir-5588 and mir-3195 at both timepoints.

To examine potential alterations of EV-miRNA profiles in detail, we performed sequencing of the isolated EV-miRNA fraction (Fig. 2A). Principal component analysis (PCA) using global expression profiles of annotated mature and hairpin miRNAs did not indicate a significant difference between samples before or after intestinal US (T1 vs. T2) (Fig. 2B). We next performed paired differential expression analysis between T1 and T2, which revealed three distinct miRNAs to be increased after intestinal US: miR-942-5p as mature miRNA as well as mir-5588 and mir-3195 as hairpin-miRNAs (Fig. 2C,D, Table 1). These novel findings hint towards specific release of EVs containing these candidate EV-miRNAs upon intestinal US.

**Table 1:** List of differentially regulated miRNA between the two timepoints in the study cohort.

Differentially regulated miRNA	BaseMean	Log2FoldChange	lfcSE	Stat	*P* value	Adjusted *P* value
Mature miRNA						
miR-942-5p	25.33299	2.23125	0.56878	3.92287	8.75 × 10^–05^	0.02223
Hairpin						
mir-5588	61.16616	1.50142	0.36804	4.07954	4.51 × 10^–05^	0.01065
mir-3195	36.08352	1.87474	0.52035	3.60286	0.00031	0.03714

### mir-942-5p induced by intestinal ultrasound in high degree acute inflammation

**Figure 3:**
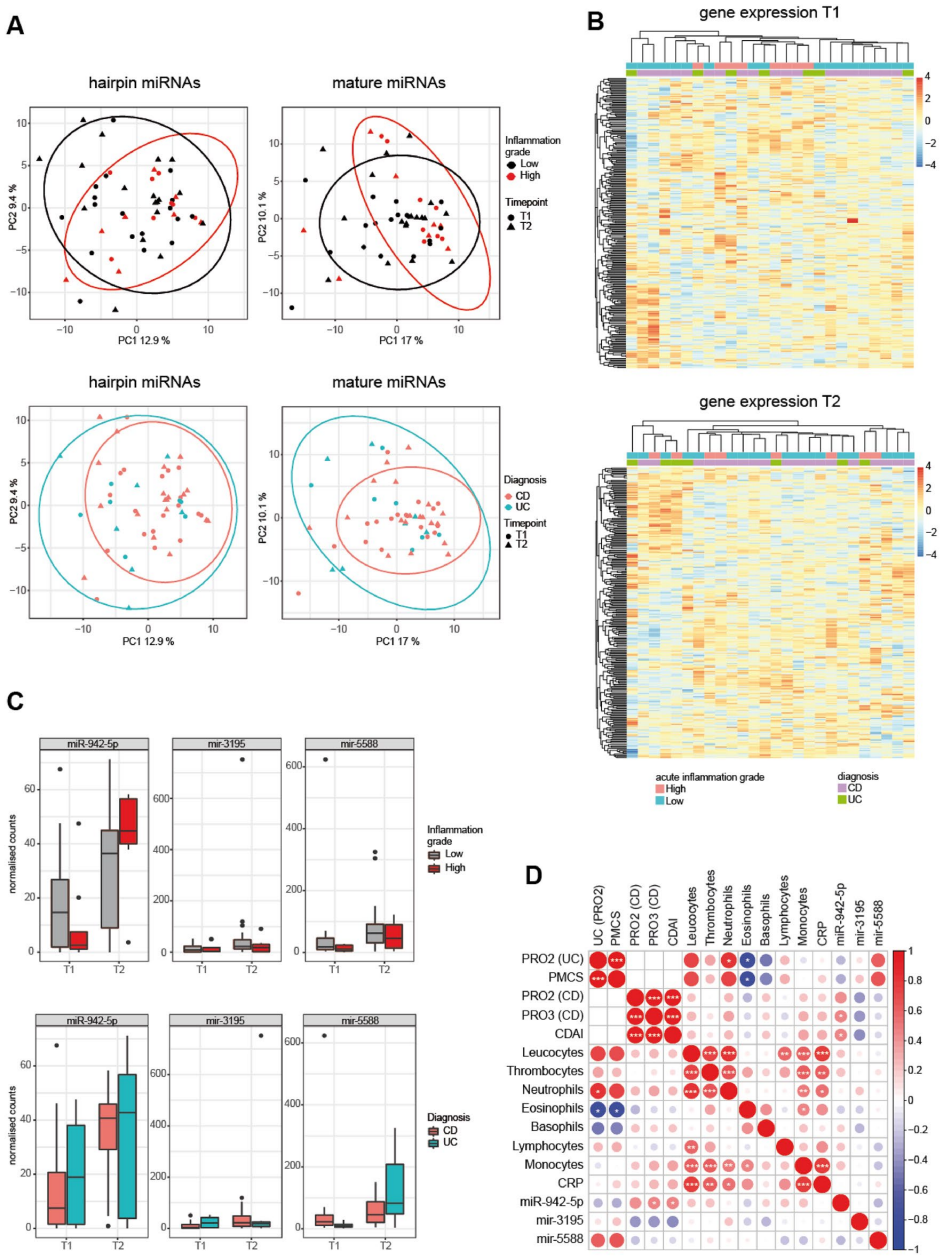
Correlation of candidate EV-miRNAs with clinical characteristics. (**A**) PCA plots of annotated miRNA, separated by US assessment of disease activity (*left*) and diagnosis (*right*). Ellipses represent 95% confidence intervals of each group. (**B**) Heatmap representing unsupervised hierarchical clustering of filtered normalized mature miRNA counts per sample at each timepoint separately, with colour coding of diagnosis and degree of acute inflammation. The color scale represents z-scores. (**C**) Normalized counts of miR-942-5p, mir-5588 and mir-3195, stratified by grade of acute inflammation (*left*) and disease entity (*right*). (**D**) Correlation matrix (Pearson correlation) between the candidate EV-miRNAs and clinical characteristics. Scale bar indicates Pearson’s rho while size of circles and white asterisks indicate *P* values (*P < 0.05, **P < 0.01, ***P < 0.001).

Next, we hypothesized that the identified inducible EV-miRNAs may correlate with clinical characteristics. Stratifying by high vs. low degree acute inflammation or by disease entity showed no significant shifts in the global miRNA expression profiles in the PCA (Fig. 3A), similarly to the analysis of the overall cohort (Fig. 2B). Furthermore, unsupervised hierarchical clustering by the EV-miRNA expression at each timepoint resulted in no clear cluster of clinical characteristics (Fig. 3B). Focusing on the three individual candidate EV-miRNAs, we observed miR-942-5p and mir-5588 to be significantly increased after US in high compared to low degree acute inflammation (Fig. 3C). We identified a significant increase of miR-942-5p after US in CD patients, while a trend towards an increase after US without reaching statistical significance was observed in UC patients. Correlating the candidate miRNAs with further clinical characteristics, we observed that miR-942-5p correlated with the clinical symptom scores in CD (CDAI, PRO3), while the two hairpin miRNAs did not significantly correlate with any disease activity scores (Fig. 3D). No significant correlation was found between the induction of the three miRNAs and CRP, thrombocytes or any immune cell type in the peripheral blood as direct or indirect markers of inflammation. In sum, induction of EV-resident miR-942-5p is associated with morphological and symptomatic signs of intestinal inflammation in Crohn’s disease.

### Regulatory function of miR-942-5p as differentially expressed EV-miRNA

Our findings demonstrated subtle, but significant changes of EV-associated miRNAs after standardized abdominal US procedure in IBD patients.

**Figure 4:**
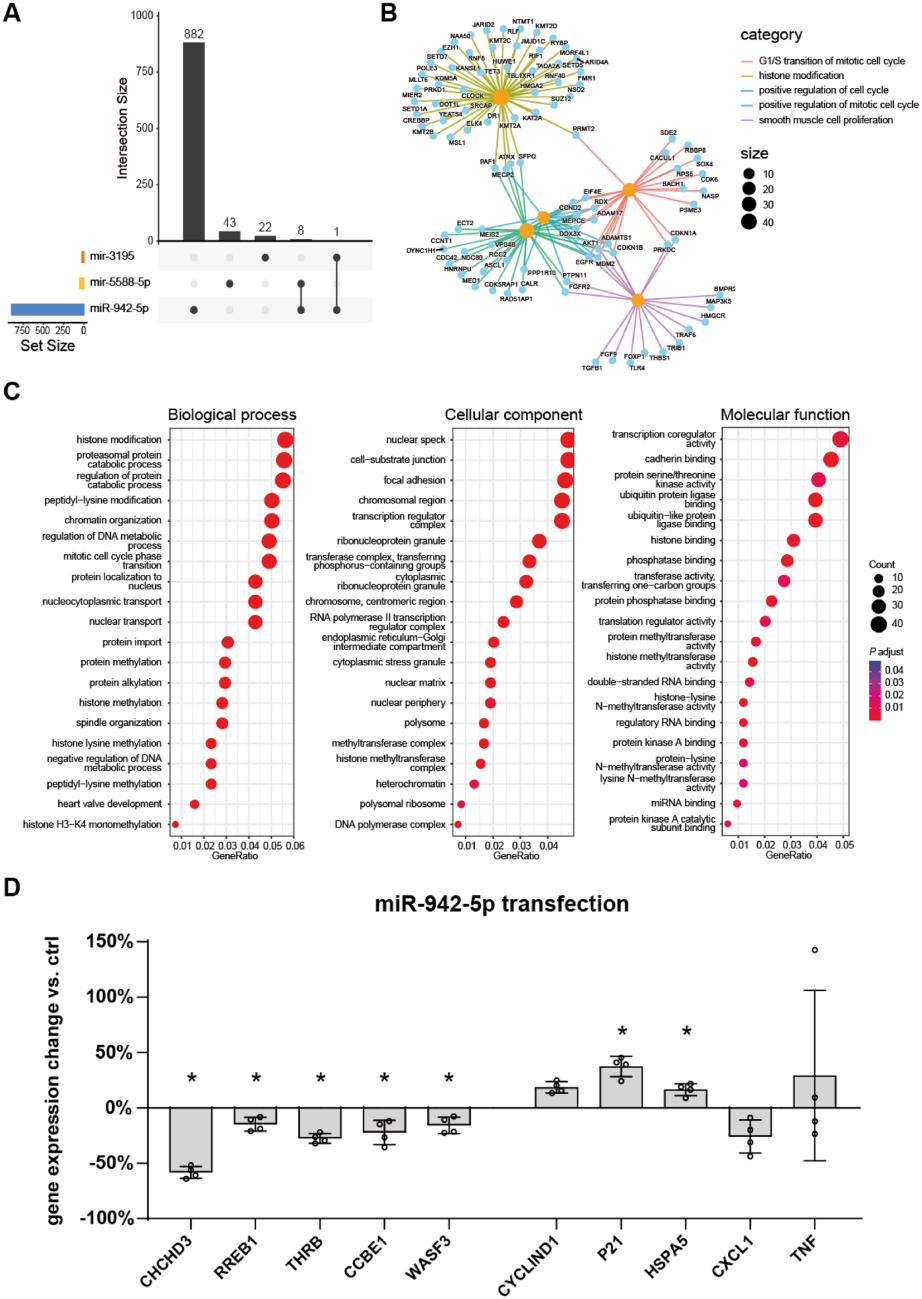
Potential functions of the differentially abundant EV-miRNAs. (**A**) UpSet plot showing genes regulated by the three candidate EV-miRNAs. (**B**) Network representation of predicted RNA targets of miR-942-5p. Genes and GO categories are depicted as nodes with different colors—orange and blue. The size of the orange-colored nodes represents the number of genes that are linked to a particular GO term. Edges are colored based on the GO categories associated with each Gene-ID. (**C**) GO term analysis of predicted target genes regulated by miR-942-5p, separated by biological processes, cellular component and molecular function. The number of genes contributing to the GO term and adjusted *P* values are represented by size of the circles and color, respectively. (**D**) Gene expression change of mRNA targets in HEK-293 cells transfected with miR-942-5p (10 nM) versus mock for 24 h (Mann–Whitney *U* test; *P < 0.05).

To assess a potential regulatory function of the three candidate miRNAs, we queried a resource for validated miRNA-target interactions. Among the three candidate EV-miRNAs, miR-942-5p is known to regulate the largest number of genes (891), while the two hairpin candidates only regulated the expression of 51 (mir-5588) and 23 (mir-3195) genes, respectively (Fig. 4A). We thus focused on the potential biological role of miR-942-5p and performed GO term analysis on the described regulated genes. The top downregulated processes were histone modification, regulation of cell cycle and WNT signaling in intestinal epithelial cells (Fig. 4B,C). This is in line with previous findings on the role of hsa-miR-942-5p in restricting colorectal cancer cell proliferation by downregulating *CCBE1*^16^. In in vitro experiments, we confirmed downregulation of *CCBE1* alongside other predicted top regulated genes by miR-942-5p in the epithelial cell line HEK-293. Conversely, expression of *CCND1*, *CDKN1A* (as cell cycle regulating genes) and *HSPA5* (as stress related gene) was increased in miR-942-5p transfected cells, while gene expression of inflammatory cytokines *TNF* and *CXCL1* was not affected (Fig. 4D).

## Discussion

Recent discoveries suggest that specific acoustic stimuli, such as LIPUS, may potently trigger the release of EVs in the context of fracture healing^17^, cancer^18^, cellular differentiation^19^ and inflammation^10,20^. Intriguingly, mechanistic work in human carcinoma cell lines hint towards calcium-dependent endosomal sorting complexes required for transport (ESCRT) pathway activation as the mode of action for exosome release upon acoustic stimulation^15^. While the concept of leveraging the specific biophysical effects of acoustic stimulation for the IBD context by dedicated procedures, such as LIPUS^21^, is followed up in larger systematic studies, we here hypothesized that local stimulation by routine abdominal sonography (even though the biomechanical properties of the US waves strongly differ from LIPUS) may also amplify the blood-based detection of EV-associated biomarkers like miRNAs, which are originating from cells in the intestinal tract. In this study, which employed 26 IBD patients of varying disease activity, we set out to identify EV-associated miRNAs, which were induced by standardized diagnostic abdominal sonography. We supposed that such miRNAs may reflect a tissue-specific signature of intestinal inflammation, which could be used to develop improved biomarkers for IBD.

Taking a pragmatic approach, we decided for a “real-world” study design with conventional transabdominal sonography devices, as potential US-induced biomarkers need to be broadly available to eventually enter clinical practice outside of specialized academic centers. The same physician performed the US investigation with the same probes and devices for all patients in the cohorts, ensuring a standardized workflow and minimized variation by the experimental design. However, we are aware of the remaining limitations of this approach. Only patients with colonic involvement (also for CD) were included in the study. It should be noted that in this pilot trial the acoustic signals as well as the physical manipulation of the tissue by the procedure itself, i.e. the pressure exerted by the sonography probe may contribute to tissue stimulation. Despite standardizing the procedure individual variation in body mass index (BMI) and concomitant abdominal wall thickness, the strength of the acoustic US signal reaching the tissue might be also variable. To identify the disease-relevant variation of induced EV-miRNAs associated with disease phenotypes, we chose a case-only design.

Using this setup, we identified a disease activity-dependent increased protein and miRNA load of EVs isolated from peripheral blood after the procedure. We detected upregulation of three EV-miRNAs (miR-942-5p, mir-5588, mir-3195) in paired post-procedural samples of IBD patients, when compared to baseline. These inducible features were more prominent in patients with a high degree of inflammatory activity. Given the short time between procedural stimulation of the tissue and sampling of peripheral blood, it is plausible that these changes reflect altered release of EVs from the intestinal tract rather than miRNA gene expression alterations. The magnitude of EV-miRNA composition changes is in line with previously described LIPUS studies in murine cell culture under well-controlled experimental conditions in vitro^10^.

Among the candidate EV-miRNAs, hsa-miR-942-5p was upregulated in high degree acute intestinal inflammation and in Crohn’s disease and has by far the largest regulatory network. hsa-miR-942-5p was described to be involved in cancer biology by interfering with cell cycle programs. However, the exact role of this miRNA in cell cycle control in colonic epithelium remained ambiguous in previously published results^16,22–25^. Our data point towards induction of cellular stress, cell cycle control and differentiation as signs of anti-proliferative effects of hsa-miR-942-5p in the context of intestinal inflammation. Further studies are also required to pinpoint the main effector cell types (epithelial vs. immune vs. stromal cells) and to elucidate the exact mechanism of immunomodulation of hsa-miR-942-5p.

In the UC group, a trend towards increased hsa-miR-942-5p expression was observed, although statistical significance was not achieved. This observation may be attributed to the relatively small sample size of the UC cohort. Consequently, while significant induction was only demonstrably evident in CD, we tentatively propose that hsa-miR-942-5p may be generally inducible in acute intestinal inflammation.

It is conceivable, however, that the detected EVs and their associated miRNAs not only reflect the local changes of the tissue but may also transmit signals to other cells or tissue compartments^9^. The cellular origin of the EV-miRNAs and the exact contribution to pathogenesis in the intestinal tissue remains to be elucidated in future functional studies.

The precipitation method used for EV isolation is known to enrich exosomes^26^. While we cannot exclude other EV types like apoptotic bodies from being in the isolates, a previous study has shown that they are unlikely to contribute considerable amounts of miRNAs^27^. The profiling of the various EV types pre- and post-US using other EV isolation methods could shed light into possible shifts in overall EV composition after acoustic stimulation of inflamed tissue. While we primarily focused on profiling of EV-miRNAs, other EV-resident molecular classes like proteins, lipids and other nucleic acids might also be altered and exert differential function in target cells^10^. Beyond EVs, cell-free DNA and RNA as further entities of liquid biopsies have been linked to IBD pathogenesis^28,29^, underpinning the need for broader and systematic molecular follow-up studies.

Potential clinical advantages of this novel type of inducible biomarkers are: (1) the sampling can be combined with routine clinical procedures, (2) they add another layer of molecular information and (3) they reflect tissue pathology better than “static” blood-based biomarkers. In this study, we correlated the induction of EV-miRNAs with disease scores and routine biomarkers at time of sampling. While inflammatory activity can be directly assessed by sonography, the additional benefit of measuring induced EV-miRNAs could be in the distinction of disease sub-phenotypes or prediction of clinical outcomes. Given the known role of miR-942-5p in colorectal cancer, it is also conceivable that US induction specifically for EV-miRNA detection methods could be a potential tool for colorectal cancer screening. While the role of (exosomal) miRNA expression already has been associated with pathogenesis^30–34^, disease phenotypes^35,36^ and therapy outcomes^37–39^ in IBD in multiple studies, the link between US-mediated induction of circulating EV-miRNAs and clinical phenotypes represents the main novelty of our work.

In conclusion, our pilot study demonstrated for the first time in a clinical setting that stimulation through conventional intestinal US can induce rapid molecular changes of EV content in the context of IBD. Despite the small cohort size, we detected distinct differentially expressed EV-miRNAs that were specifically upregulated after US and in high degree acute intestinal inflammation. These pilot results warrant further investigations into the biological function of differentially expressed EV-miRNA, other EV biomolecule classes (e.g. by utilizing proteomics approaches) and larger controlled studies validating EV associated inducible miRNAs as biomarkers for disease activity and therapeutic response.

## Materials and methods

### Study cohort

From September 2020 to January 2021, 26 IBD patients (19 with CD and 7 with UC) with an indication for clinical monitoring via transabdominal US were enrolled at the University Medical Center Schleswig–Holstein, Kiel Campus. Patients with any disease activity were included, while a definite diagnosis of either Crohn’s disease (with colonic involvement = L2 or L3 according to Montreal classification) or ulcerative colitis (at least with left-sided colon = E2 or E3 according to Montreal classification) was required, ensuring colonic involvement in all enrolled patients. Patients with any approved maintenance therapy were included, with the restriction that the medication and symptoms needed to be stable for 3 months prior enrollment. Induction therapies or new medications within the last 3 months as well as previous intestinal surgery were exclusion criteria. The recruitment was restricted to the availability of one experienced sonographer to minimize inter-operator bias of disease activity. This prospective clinical study concomitant to the clinical routine was approved by the ethics committee of the Christian Albrecht University of Kiel (D 556/20) in accordance with the Declaration of Helsinki and all enrolled subjects provided written informed consent. All the following experimental and analytical procedures were carried out in accordance with relevant guidelines and regulations. Blood samples in serum containers were obtained before and 30 min after the start of the diagnostic intestinal US procedure. The timepoint was chosen based on previously described peak timepoints for US/LIPUS induced EV secretion^15^. Demographics, baseline characteristics, medication, disease activity scores as well as details of the US diagnostics (location and severity of inflammation) were recorded (Table 2). Status of acute inflammation was classified in CD as “high” (severe/moderate acute inflammation) according to a Simple Ultrasound Activity Score for CD (SUS-CD) ≥ 3^40^ versus “low” (mild/no signs of acute inflammation) grade (SUS-CD < 3). In adaptation to the CD criteria and the Milan Ultrasound Criteria^41^, presence of bowel wall thickening ≥ 4.5 mm and hypervascularization in UC patients led to the classification of “high grade” disease activity. These thresholds were chosen based on previously described correlation with moderate-to-severe endoscopic activity.

**Table 2:** Demographics and clinical characteristics of patients with IBD who were included in this study.

	IBD patients [*n* = 26]
Age (years), median [range]	32 [19–73]
Female sex [%]	14 [53.8]
CRP (mg/L): median [range]	5.45 [0.74–41.7]
Leucocytes (cells/nL): median [range]	7.51 [4.09–15.93]
BMI (kg/m^2^): median [range]	22.8 [17.2–33.2]
Subtype of IBD [%]	
CD	19 [73.1]
UC	7 [27.9]
Location of disease: CD [% of total CD]	
L2	8 [42.1]
L3	11 [57.9]
Location of disease: UC [% of total UC]	
E2	5 [71.4]
E3	2 [28.6]
Biologics therapy [%]	
Anti-TNF	14 [53.8]
Vedolizumab	1 [3.8]
Ustekinumab	7 [26.9]
None	4 [15.3]
Corticosteroid use [%]	
Prednisolone oral (≤ 5 mg/d)	2 [7.7]
Budesonide oral	3 [11.5]
Budesonide rectal	1 [3.8]
Immunomodulators [%]	
Azathioprin	2 [7.7]
Mesalamine oral	7 [26.9]
Crohn’s disease activity index (CDAI, CD CD): median [range]	64 [16–262]
Partial mayo clinical score (PMCS, UC): median [range]	4 [1–8]
Maximal bowel wall thickness (mm): median [range]	4.25 [2–8]
Sonographic grade of acute inflammation	
High grade [%; CD/UC]	8 [30.8; 5/3]
Low grade/remission [%; CD/UC]	18 [69.2; 14/4]

### Transabdominal ultrasound

All sonography procedures in this study were performed using the same sonography device (Logiq E9, GE Healthcare, Chicago, IL) and the same linear array probe (9L-D, GE Healthcare, Chicago, IL). The procedure was performed in a standardized fashion, starting with the assessment of the sigmoid colon and continuation of US scanning in an aboral-to-oral direction. By assessing sonographic findings like vascularity and gut wall thickness, active intestinal inflammation was graded in high-grade vs. low-grade acute inflammation/remission^2,42^. All ultrasound procedures were performed by one board certified gastroenterologist and experienced intestinal sonographer.

### Isolation and quantification of serum EVs

Blood collected in serum blood containers were centrifuged at 2000×*g* for 10 min at room temperature. Serum EVs were then isolated employing two different isolation methods: (1) via precipitation using “ExoQuick™—Exosome Precipitation Kit” (#EXOQ20A-1, Biozol, Eching, Germany) according to the manufacturer’s protocol from 500µL aliquots. This method was chosen for miRNA isolation as it increases the yield of miRNA, but with limitations for quantification of size and numbers of EVs. (2) via serial centrifugation at 4 °C eliminating cell debris and apoptotic bodies in two cleaning steps (30 min at 300×*g* and 30 min at 2000×*g*), the supernatant was centrifuged before at 12000×*g* for 45 min and then at 100,000×*g* for 90 min, yielding non-precipitated EVs applicable for the following downstream analyses. The quantification of EVs and the size distribution are defined and analysed with Nanosight NS300 Nanoparticles Tracking Analysis (NTA)-based on the number of vesicles per mL. Detection of EV surface proteins CD9, CD63 and CD81 was performed on a fraction of available samples (30 samples from 15 patients) using the MACSPlex EV KIT IO (human, #130-108-813, Miltenyi Biotech, Bergisch-Gladbach, Germany) according to the manufacturer’s protocol and measurement was performed via flow cytometry using the LSRFortessa™ (BD Biosciences, Franklin Lakes, NJ, USA) to detect the EV-bound proteins CD1c, CD2, CD3, CD4, CD8, CD9, CD11c, CD14, CD19, CD20, CD24, CD25, CD29, CD31, CD40, CD41b, CD42a, CD44, CD45, CD49e, CD56, CD62P, CD63, CD69, CD81, CD86, CD105, CD133/1, CD142, CD146, CD209, CD326, HLA-ABC, HLA-DRDPDQ, MCSP, ROR1 and SSEA-4.

### Small RNA extraction and miRNA quantification

50µL of this suspension were subjected to small RNA extraction using of the “SeraMir™ Exosome RNA Amplification Kit” (#RA808A-1, Biozol, Eching, Germany) with an adapted protocol for extracellular RNA isolation^43^. The isolates were eluted in 30µL elution buffer and miRNA concentration was quantified using the “Qubit™ microRNA Assay Kit” (#Q32880, Invitrogen™, Waltham, MA) according to the manufacturer’s protocol. Samples with a concentration below the lower limit of detection (500 ng/mL serum) were assumed to have a concentration of half the lower limit of detection (250 ng/mL serum) as this method introduces least bias^44^.

### miRNA sequencing

miRNA-sequencing libraries were prepared according to the manufacturer’s protocol of the NEXTFLEX^®^ Small RNA-Seq Kit v3 (#NOVA-5132–06, PerkinElmer, Waltham, MA) and sequenced on SP lanes on an Illumina NovaSeq 6000 (2 × 50 bp).

### Computational processing

Reads were processed by the nf-core/smrnaseq(v 1.1.0) pipeline^45^. In summary, reads were trimmed with Trim Galore! (v 0.6.5). Reads were first clipped by 4 bases on the 5′ and 3′ ends followed by adapter trimming. Reads were then mapped to annotated miRbase (release 22) miRNAs with Bowtie1 (v 1.3.1)^46^. Reads were first mapped to annotated mature miRNAs with unmapped reads subsequently mapped to annotated hairpin miRNAs. Count estimation was done using Samtools (v 1.12)^47^.

### Downstream analysis

All count analysis was conducted in R 4.0.5 (R Core Team, 2021). Mature and hairpin counts were processed separately. Multiple detection thresholds were tested to filter miRNAs that are robustly detected within the cohort, e.g. more than 5 counts in 25% of samples as described before^48^ or at least 1 count in 90% of samples. In the downstream analysis, the latter setting yielded lower numbers of differentially expressed miRNAs, which were all also identified in the first setting (> 5 counts in 25% of samples). To have more robust results, the setting with at least 1 count in 90% of samples was chosen. Variance stabilizing transformation was applied to raw counts via DESeq2 (v 1.3.0), with normalized counts being used for each principle component analysis^49^.

Differential expression analysis was subsequently performed on the filtered reads with DESeq2 (v 1.3.0). Counts were modelled with both patient and time point (~ patient + time point) using a negative binomial model with shrunken dispersion estimates determined using a local fit method. Differentially expressed miRNAs between pre- and post-induction time points were determined at an alpha of 0.05 with Benjamini and Hochberg adjusted *P* values.

For unsupervised clustering, normalised mature miRNA counts at each sampling time point, were Z-score normalised by row and hierarchically clustered using pheatmap (v 1.0.12).

### Assessment of predicted genes regulated by candidate miRNAs

To identify the validated target of each miRNA, we used multiMir (v1.16.0)^19^ R package under default settings (summary = TRUE) inside R version 4.1.3 (R Core Team, 2022). multiMir uses multiple external databases, including miRecords, miRTarBase, and TarBase to identify predicted and validated miRNA-target interactions^50^, while for downstream analysis only experimentally validated miRNA-target interactions were used. Next, we used clusterProfiler^51^ (v.4.2.2) R package for the functional analysis of the identified target genes, including cellular component (CC), biological process (BP), and molecular function (MF). To display the top 20 enriched GO terms for each ontology, we used the dotplot function in clusterProfiler (v.4.2.2). To display the network of selective GO terms with multiple genes, we utilized the cnetplot function.

Upset plot was used to show the intersection of target genes among multiple miRNAs using VennDetail^52^ (v.1.14.0) R package.

### Lowry assay

For protein extraction of the precipitated EV pellets was suspended in 100 µL of RIPA lysis buffer (#89900, ThermoFischer, Waltham, MA) with 1% Halt™ Proteinase Inhibitor Cocktail (#78430, ThermoFischer, Waltham, MA) and incubated at 4 °C for 30 min while agitating. Samples were sonicated twice for 5 s before being centrifugated at 16000×*g* for 15 min at 4 °C. Supernatants were subjected to Lowry assay using the “DC Protein Assay Kit II” (#5000112, BioRad, Hercules, CA) according to manufacturer’s protocol.

### miRNA transfection and qPCR

For miRNA transfection experiments, HEK-293 cell line (ACC305, DSMZ, Braunschweig, Germany) cultured in Dulbecco’s Modified Eagle’s Medium (Gibco, Waltham, MA) + 10% FBS at 37 °C and 5% CO_2_ and seeded out in a concentration of 10,000 cells /24-well on the previous day were transfected with 10 nM hsa-miR-942-5p (mirVana™ miRNA mimic, MIMAT0004985; Ambion, Waltham, MA) or mock using Lipofectamine™ RNAiMAX reagent (#13778075, ThermoFischer, Waltham, MA) for 24 h. RNA was subsequently isolated using RNEasy Mini Kit (#74104, Qiagen, Hilden, Germany) and cDNA was generated using RevertAid Premium cDNA Synthesis kit (#K1622, ThermoFischer, Waltham, MA) according to manufacturer’s protocol. Gene expression was subjected to the cDNA samples using SYBR Green qPCR. Reactions were performed on the ViiA™ 7 Real-Time PCR System (Applied Biosystems, Waltham, MA), and relative gene expression levels were determined using *ACTB* as a housekeeper. Primer sequences were either retrieved from PrimerBank^53^ or from previous publications or designed using Primer3 software version 0.4.0^54^ and are listed below.

**Table:** 

Gene	Primer sequence (forward, reverse)	Source
*SLC4A10*	5′-GTCACAGGCATCGTGGTCATA-3′ 5′-CCTTCACGCCAACAAATCTCAT-3′	PrimerBank, ID: 295821222c1
*CHCHD3*	5′-GAGGCGGACGAGAATGAGAAC-3′ 5′-ACCAGAATACCGCTGAGACTTC-3′	PrimerBank, ID: 142365297c1
*SGMS1*	5′-TGTGCCGAGTCTCCTCTGA-3′ 5′-CCGTTCTTGTGTGCTTCCAAA-3′	PrimerBank, ID: 41350331c1
*RREB1*	5′-AGGTTCAGACCTATCTTCCATCA-3′ 5′-CTGCCAATCCGATTTGGTCCT-3′	PrimerBank, ID: 270132928c1
*KNG1*	5′-TGCTCCAGGCTGCTACTAAGT-3′ 5′-GGCTTCAGTTATGCGGTACAA-3′	PrimerBank, ID: 262050545c1
*DIAPH2*	5′-TTACCACCAAACGTGAGATGG-3′ 5′-CGGATTGCTGGTTAAAGAAACCC-3′	PrimerBank, ID: 189242611c1
*LPP*	5′-CCCGGTAGTTGCTCCAAAAC-3′ 5′-CCCTGTACTTTGAAAGCCTCTTC-3′	PrimerBank, ID: 264681525c1
*RANBP3L*	5′-AAATCTGTCATTGCTCAACCCA-3′ 5′-GCTGCTTCATACAGGGTGTCTT-3′	PrimerBank, ID: 239582742c1
*THRB*	5′-TGGGACAAACCGAAGCACTG-3′ 5′-TGGCTCTTCCTATGTAGGCAG-3′	PrimerBank, ID: 358001056c1
*HSPD1*	5′-ATGCTTCGGTTACCCACAGTC-3′ 5′-AGCCCGAGTGAGATGAGGAG-3′	PrimerBank, ID: 41399283c1
*RND3*	5′-GCTCCATGTCTTCGCCAAG-3′ 5′-AAAACTGGCCGTGTAATTCTCA-3′	PrimerBank, ID: 362999163c1
*CCBE1*	5′-CGACTAAATACCCGTGTCTGAAG-3′ 5′-TCGGCACAAACGTCGTAATCT-3′	PrimerBank, ID: 290562712c1
*WASF3*	5′-AAGGGATTACCAGCGAACTTG-3′ 5′-CTTCAGCATGTTTGCTCAGACT-3′	PrimerBank, ID: 221316712c1
*CXCL1*	5′-AACCGAAGTCATAGCCACAC-3′ 5′-GTTGGATTTGTCACTGTTCAGC-3′	Wang et al.^55^
*TNF*	5′-CTTCTGCCTGCTGCACTTTGGA-3′ 5′-TCCCAAAGTAGACCTGCCCAGA-3′	Cowan et al.^56^
*HSPA5*	5′-GACATCAAGTTCTTGCCGTT-3′ 5′-CTCATAACATTTAGGCCAGC-3′	Primer3
*CCND1*	5′-ACTACCGCCTCACACGCTTC-3′ 5′-CAGGTCCACCTCCTCCTCCT-3′	Primer3
*CDKN1A*	5′-AGAGGAGGCGCCATGTCAG-3′ 5′-CGTGGGAAGGTAGAGCTTGG-3′	Primer3

### Statistics

Statistical analysis was performed using the GraphPad Prism 8 software package (GraphPad Software Inc., La Jolla, CA) and R 4.0.5 (R Core Team, 2021). The used statistical tests and levels of significance were chosen for each experiment as appropriate and are indicated at each figure.

## Data Availability

The raw and processed miRNA-sequencing data generated during this study is available and is accessible at the NCBI GEO website under the accession number GSE237994.
